# An example of phenotypic adherence to the island rule? – Anticosti gray jays are heavier but not structurally larger than mainland conspecifics

**DOI:** 10.1002/ece3.1557

**Published:** 2015-08-14

**Authors:** Dan Strickland, D Ryan Norris

**Affiliations:** 1 1063 Oxtongue Lake Road, Dwight, Ontario, P0A 1H0, Canada; 2 Department of Integrative Biology, University of GuelphGuelph, Ontario, N1G 2W1, Canada

**Keywords:** Biogeography, intraspecific competition, islands, *Perisoreus canadensis*

## Abstract

The island rule refers to the tendency of small vertebrates to become larger when isolated on islands and the frequent dwarfing of large forms. It implies genetic control, and a necessary linkage, of size and body-mass differences between insular and mainland populations. To examine the island rule, we compared body size and mass of gray jays (*Perisoreus canadensis*) on Anticosti Island, Québec, located in the Gulf of St. Lawrence, with three mainland populations (2 in Québec and 1 in Ontario). Although gray jays on Anticosti Island were ca 10% heavier, they were not structurally larger, than the three mainland populations. This suggests that Anticosti jays are not necessarily genetically distinct from mainland gray jays and that they may have achieved their greater body masses solely through packing more mass onto mainland-sized body frames. As such, they may be the first-known example of a proposed, purely phenotypic initial step in the adherence to the island rule by an insular population. Greater jay body mass is probably advantageous in Anticosti's high-density, intensely competitive social environment that may have resulted from the island's lack of mammalian nest predators.

## Introduction

Small mammals established on islands tend to become structurally larger and heavier than their mainland ancestors, whereas insular populations of larger species (>100 g) often develop dwarf forms (Foster 1964), a pattern termed the “island rule” by Van Valen (1973). Case (1978) proposed that insular body-size trends might be explained by ecological release from predation, parasitism, and interspecific competition and/or by intense intraspecific competition for limited resources exacerbated by high densities of conspecifics. Other workers expanded this perspective predicting that, following ecological release, evolution would tend towards taxon-specific optimum sizes variously estimated to be, for mammals, 100 g (Brown et al. 1993), 1000 g (Damuth 1993), or, for birds, 33 g (Maurer 1998). While the existence of optimum body sizes on islands has been challenged (Raia et al. 2010), the general validity of the island rule has been supported for mammals using increasingly larger datasets notably by Lomolino (1985, 2005), Lomolino et al. (2010, 2012, 2013), and Palombo and Rozzi (2013). It has also been extended to other taxa, although this has been disputed for some groups (e.g., turtles; Itescu et al. 2014) and even for mammals (Meiri et al. 2006, 2008).

Some early investigators reported longer bills and tarsi in island birds but doubted there was consistent avian adherence to the island rule (Amadon 1953; Grant 1965, 1968). Nevertheless, Clegg and Owens (2002) and Lomolino (2005) later affirmed the island rule in birds, particularly in initially large nonpasserines, and numerous cases of larger-than-mainland body sizes are also known in island passerines. The examples include north Atlantic island populations of the wren (*Troglodytes troglodytes*; Williamson 1981), the Gotland population of the coal tit (*Periparus ater*; Alatalo and Gustafsson 1988), the island scrub-jay (*Aphelocoma insularis*) of Santa Cruz Island CA, (Curry and Delaney 2002), the Capricorn white-eye (*Zosterops lateralis chlorocephalus*) of Heron Island, Australia (Robinson-Wolrath and Owens 2003), and by several island populations of the savannah sparrow (*Passerculus sandwichensis;* Wheelwright and Rising 2008). Amadon (1953) reported that passerines predominated among the ca 20 Fernando Po bird species that were larger than their African mainland counterparts.

There is almost no mention of body masses (as opposed to body “sizes”) in the literature on avian adherence to the island rule, and the strong implication seems to have been that differences in size and mass are necessarily two sides of the same coin. Grant made this explicit, stating “*the most satisfactory measure of body size is fat free body weight…”*, before pointing out that body weight had rarely been determined for island birds and resorting, with caveats, to the use of wing length as a “size” index (Grant 1965). In contrast, different insular body “sizes” in mammals are typically reported as differences in body mass, with linear measures such as body or skull length used only when mass data are unavailable (e.g., Meiri and Dayan 2003; Lomolino 2005).

Differences in structural body size imply genetic control and this has been shown in the Gotland coal tits (Alatalo and Gustafsson 1988). Since adherence to the island rule is typically perceived to involve differences in both body size and body mass, there is an implicit suggestion that mainland–island body mass differences are likely also under genetic control. Grant (1990) nevertheless wondered if “*differences between island and mainland features [might] be simply different phenotypic expressions of the same genotypes raised under different environmental conditions*.” Adler and Levins (1994) proposed further that features of the “island syndrome” in rodents, including body “size,” could be initial, purely phenotypic responses to higher island densities that might be subsequently followed by actual selection for larger body “sizes.” If this is true, the possibility is raised, at least in birds, that mainland–island body-mass differences might not always be associated with differences in structural body size given that clearly phenotypic, “constant-size” mass changes are well known in different avian contexts. These include changes in “fatness” (Ekman and Hake 1990), the 25% or greater increases observed in some prelaying females (Sechley et al. 2013 and references therein), the 10% daily increases achieved before sunset by some boreal wintering passerines (Haftorn 1992), and the maintenance of greater body weights over longer periods by subordinate individuals of other species, apparently as a hedge against being excluded from food sources by more dominant conspecifics (Ekman and Hake 1990; Clark and Ekman 1995; Pravosudov et al. 1999).

**Figure 1:**
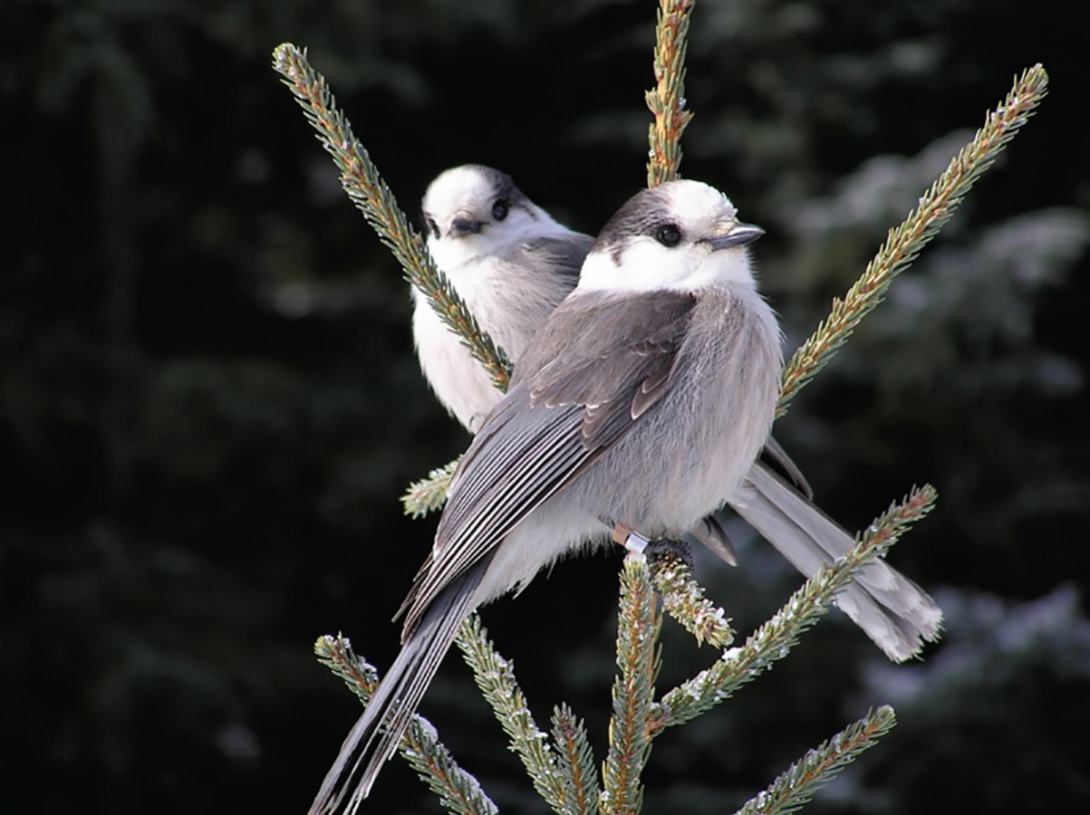
A male and female breeding pair of gray jays (*Perisoreus canadensis*) in Algonquin Park, Ontario, Canada. Credit: Dan Strickland.

We undertook this study after discovering that mean weights of gray jays (*Perisoreus canadensis*; Fig.1) on Anticosti Island, Québec, (males 82.7 g, females 74.9 g; Strickland and Ouellet 2011) are ca 10% greater than in our long-term study population in Algonquin Park, Ontario (males 75.4 g, females 67.5 g). We asked, first, whether similar differences exist between Anticosti gray jays and the two mainland Québec populations (both south and north) closest to the island. Finding that the island jays do indeed have greater body masses than all three mainland populations, we expected to find corresponding mainland–island differences in linear measurements, albeit small ones as they would presumably scale as the cube root of the mass differences. We therefore compared mainland–island mass and structural size differences with those of males versus females and, in conjunction with other work on Anticosti, collected demographic data, particularly population density, that might shed light on why Anticosti gray jays are heavier but not structurally larger than their mainland counterparts.

## Materials and Methods

### Study areas

**Figure 2:**
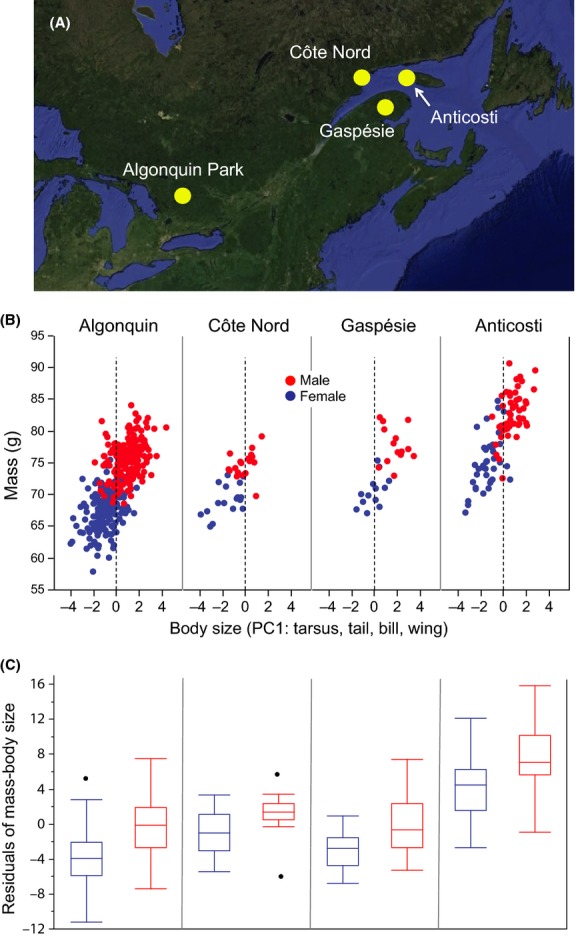
Variation in body (structural) size and mass among four gray jay populations. (A) map of study area showing the four study sites, (B) The relationship between body size (first principal component scores of tarsus, bill length, and wing) and mass among the four populations and broken down by sex (see Results for model details), (C) Box plots showing the relationship between the residuals of mass–body size and sampling site, broken down by sex (see Results for model details). Black dots above or below box plots identify outliers.

Québec's Anticosti Island (49°N, 63°W; 7943 km^2^; hereafter called “Anticosti”) is in the Gulf of St. Lawrence, ca 30 km from the Côte Nord (north coast) at the closest point, 60 km from the Gaspé Peninsula to the south, and 250 km from Newfoundland to the east (Fig.2A). Most of the land is covered by boreal forests of white (*Picea glauca*) and black spruce (*Picea mariana*) although there are also many large, treeless bogs, and small lakes. High densities of introduced white-tailed deer (*Odocoileus virginianus*) have eliminated most ground flora and are destroying the balsam fir (*Abies balsamea*) component of the original forest (Potvin et al. 2003). The avifauna of Anticosti most closely resembles that of the Gaspé Peninsula or southwest Newfoundland (Ouellet 1969), but the density of gray jays on Anticosti was deemed greater than in any other area covered by the 1984–1989 breeding bird atlas of Québec (Gauthier and Aubry 1996).

Gray jays from three mainland areas were also sampled (Fig.2A): Algonquin Provincial Park, Ontario (45°50′N, 78°20′W; hereafter called “Algonquin”), along the Route du Lac Ste Anne, south of Gaspésie Park, Québec (48°55′N, 65°50′W; hereafter called “Gaspésie”), and along the SM-3 power station road, west of Sept-Îles, Québec (50°20′N, 64°28′W; hereafter called “Côte Nord”). Descriptions of the climate, physiography, forest composition, and local avifaunas of the Quebec study areas are available in Gauthier and Aubry (1996) and for the Algonquin Park study area in Strickland (1991).

### Data collection

We banded, weighed, measured, obtained blood samples from, and recorded GPS locations for, 106 adult or first-year Anticosti gray jays within 15 km of the village of Port Menier during the following intervals: October 18–November 14, 2001; March 1–June 11, 2002; and October 26–28, 2002. To assure seasonal comparability with other sites, for this analysis, we retained data from 85 of these jays (45 males, 40 females), all handled in the period October 15–November 15. Similar data were obtained from 27 jays (14 males, 13 females) in Gaspésie October 21–23, 2002, and from 35 jays (18 males, 17 females) on the Côte Nord, October 29–November 1, 2002. Weights and linear measurements were obtained from 224 individuals (115 males, 109 females) in Algonquin during October 15–November 15 from 1979 to 2013.

In all areas, we attracted jays to temporary suet feeding stations, captured them in Potter traps, and then immediately banded, weighed, and measured them, releasing them 5 min or less after capture. All individuals were banded with standard numbered aluminum bands and, on Anticosti and in Algonquin, two or three colored bands to facilitate later behavioral observations as part of another study (Fig.1). Weight was estimated to the nearest 0.5 g before 1994 and to the nearest 0.1 g afterward with a 100 g Pesola scale. Bill length was measured by placing one tip of pointed metal dividers snugly against the inside anterior edge of the nasal aperture, adjusting the other tip so that it made contact with and barely brushed by the tip of the maxilla, and then measuring the separation so obtained to the nearest 0.1 mm against a notched ruler. Tarsus length was similarly estimated by placing one divider tip in the “notch” exposed at the proximal end of the tarsus with the leg flexed, and the other tip at the distal end of the tarsus (with the foot flexed). Length of the seventh primary was estimated by placing one end of the dividers snugly against the manus between the seventh and eighth primaries and adjusting the other end of the dividers so that its tip touched the distal end of the seventh primary. Wing chords and tail lengths were obtained using the ruler directly (*i.e*., without the dividers). Blood samples were obtained by piercing the brachial vein at the distal humeral joint, filling a capillary tube, and transferring the blood to preservative-treated paper.

### Sex determination

Algonquin jays were sexed by observing female-specific behavior (incubation) in color-banded pairs. Jays from Anticosti, Gaspésie, and the Côte Nord were sexed from red blood cell chromosomes at the Wildlife Forensic DNA Laboratory of Trent University, Peterborough, ON (courtesy of Angela Coxon and B.N. White), using polymerase chain reaction (PCR) with avian gender primers 2718-R and 2550-F (Griffiths et al. 1998). Using these primers, a dilution series was prepared to determine the optimal reaction concentration, and a gradient was run to determine the optimal annealing temperature for the sexing reaction (55°C).

### Estimation of population density on Anticosti Island

We estimated territory size for Anticosti gray jays using the mean of nine internest distances obtained by a handheld GPS unit in 2002, and the formula (3/2) *d*^2^tan30 where *d* is the distance between centers of adjacent equal hexagons (Strickland and Ouellet 2011). We then calculated the density of territories and, using the observed ratio of breeders to nonbreeders, a population density that we were able to compare with similar estimates from mainland areas.

### Data analysis

We ran two general linear models: the first to explain variation in structural size and the second to explain variation in weight. To first obtain an estimate of structural size, we ran a principal components analysis on the correlations (estimated by restricted maximum likelihood) of bill length, tarsus length, and wing chord (*n *=* *361). The eigenvectors for all three variables were fairly high, and all loaded positively on the first principal component (PC1) score (bill: 0.56, tarsus: 0.62, wing chord: 0.54), explained 58% of the cumulative variation. For the structural size model, we included sex, study area, and study area*sex as predictor variables. For the weight model, we used these same predictor variables but added structural size (PC1 scores from the analysis above) because we were interested whether weight varied by study area or sex while controlling for structural size. For both models, alpha level was set at 0.05 and all analyses were performed in JMP Statistical Software v11.2.0, SAS, Cary, NC.

## Results

### Structural size and mass

Structural size, as estimated from PC1 scores of tarsus, bill length, and wing chord, varied by study area but not in the direction predicted by the island rule. A model with study area, sex, and study area*sex explained 54% of the variation in structural size (first principal component score; *R*^2^* *=* *0.54, *F*_7,353_ = 59.8, *n *=* *361, *P *< 0.0001). Both study area (*F *=* *18.6, *P *<* *0.0001; Fig.2B) and sex (*F *=* *173.4, *P *<* *0.0001; Fig.2B) were highly significant, but the interaction was not (*F *=* *3.0, *P *=* *0.38). Gray jays from Gaspésie (least squares mean ± SE: 0.79 ± 0.19) tended to be structurally larger than jays from Algonquin (0.08 ± 0.07), Anticosti (−0.33 ± 0.11), and Côte Nord (−0.92 ± 0.17) and, as expected, males (−0.85 ± 0.10) were larger than females (−1.04 ± 0.10).

In contrast, variation in mass while controlling for structural size tended to follow the pattern of the island rule. A model with structural size, study area, sex, and study area*sex explained 76% of the variation in mass (*R*^2^ = 0.76, *F*_8,351_ = 141.2, *n *=* *360, *P* < 0.0001). Structural size (*F *=* *40.1, *P *<* *0.0001), study area (*F *=* *135.5, *P *<* *0.0001), and sex (*F *=* *114.2, *P *<* *0.0001; Fig.2C) were highly significant, but the interaction was not (*F *=* *1.2, *P *=* *0.32). Anticosti birds (least squares mean ± SE: 79.3 ± 0.3) tended to be heavier while controlling for structural size than Gaspésie (73.3 ± 0.6), Côte Nord (72.8 ± 0.5), or Algonquin (71.7 ± 0.2) birds, and males (77.0 ± 0.3) were heavier while controlling structural size than females (71.6 ± 0.3).

### Population density

Based on a mean internest distance of 561 m (*n *=* *9), we estimated the mean territory size of gray jays on Anticosti as 27.3 ha. This is much lower than corresponding estimates of territory size from Réserve de la Vérendrye, QC (69 ha; Strickland and Ouellet 2011), Algonquin Park (146 ha; Strickland and Ouellet 2011), Manitoba (65 ha; Walley 1981), and the Yukon (41 ha; Shank 1986). These estimates convert to: 3.7 (Anticosti), 1.4 (la Vérendrye), 0.7 (Algonquin), 1.5 (Manitoba), and 2.4 (Yukon) territories per 100 ha. Accounting for available fall nonbreeder-pair ratios (Anticosti: 0.80, this study, la Vérendrye: 0.42, Strickland and Ouellet 2011; Algonquin 0.43, D. Strickland, unpubl. data), we estimated the corresponding fall density on Anticosti to be 10.3 jays/100 ha), that is, much greater than in la Vérendrye (3.5 jays/100 ha) or in Algonquin (1.7 jays/100 ha).

## Discussion

The island rule is usually described as involving an insular species or subspecies that differs in “size” from the corresponding mainland taxon. Given the usually assumed necessary coupling of structural size and mass (e.g., Grant 1965), the expectation is that an insular form conforming to the island rule should be both structurally larger and correspondingly heavier than the related mainland form. We found, however, that gray jays on Anticosti Island were ca 10% heavier, but not structurally larger, than three mainland populations. Moreover, if Anticosti and mainland jays differed at all in structural size, it was in the direction opposite to that predicted by the island rule (i.e., one mainland population, while having significantly larger structural sizes compared to Anticosti birds, nevertheless had much lower body masses; Fig.2B).

In contrast to these island–mainland patterns, males in all four populations were not only heavier than females (by a ca 10% margin, similar to the Anticosti–mainland body-mass difference) but were also structurally larger (Fig.2B and C). The larger size of males is obviously under genetic control, but our failure to find larger body sizes in the heavier-than-mainland jays on Anticosti suggests that they may not be genetically distinct from mainland birds. Instead, Anticosti gray jays may be conforming to the island rule solely through phenotypically packing more tissue on more or less mainland-sized body frames. Of course, it is also possible that selection has favored an increased ability on the island to facultatively elevate body masses in response to some feature of the Anticosti environment. It remains unknown, therefore, whether mainland birds transported to Anticosti would achieve any body mass increases or, if so, whether they would be comparable to those we found in the Anticosti population. A recent phylogeographic analysis of the gray jay (van Els et al. 2012), while revealing surprising genetic variability in the major taiga clade occupying all suitable habitat in eastern and northern Canada, nevertheless failed to detect genetic differences between Anticosti and Québec mainland jays (respectively referred to as “Côte Nord” and “Saguenay” in their survey; P. van Els, pers. comm.). This phenotypic hypothesis also supports previous rejections (Taverner 1937; Ouellet 1969) of the subspecific status (*Perisoreus canadensis barbouri*) once accepted for Anticosti gray jays (Anonymous 1931, 1957).

We do not know when gray jays colonized Anticosti although it could have been as long ago as 4000–8500 B.P. with the establishment of the island's first postglacial spruce forests (Lavoie and Filion 2001). Gray jays are slow-flying, nonmigratory occupants of permanent boreal forest territories and only rarely irrupt southwards (Strickland and Ouellet 2011). The birds involved in these exceptional movements (mostly or even exclusively juveniles, unpubl. data) may concentrate in small flocks when they encounter large, east–west-aligned water bodies such as the Gulf of St. Lawrence (Todd 1963; Campbell 1965), and the colonization of Anticosti plausibly occurred when such a flock was blown out to sea and managed to land on the island 30 km to the south. As we estimate Anticosti's gray jay population to be on the order of 25,000 (unpubl. data), it is doubtful that possible repeat arrivals from the Côte Nord have any significant genetic impact on the island jays and it is far less likely northward flights from the island even occur, let alone influence the Côte Nord population. Nevertheless, the lack of Anticosti–mainland genetic differentiation (van Els et al. 2012) suggests either that there is significant mainland-to-island gene flow or that the founding event occurred relatively recently. Whatever the case, while a selective pressure for greater body mass may now be operating on the island, Anticosti gray jays do not presently exhibit a genetically controlled increase in structural size. Instead, following the scenario proposed by Adler and Levins (1994), they may be initially responding to the Anticosti environment solely through a ca 10% phenotypic mass increase. Much greater phenotypic mass increases in gray jays within a season have been documented elsewhere (e.g., mean weight gains of 28% in just 9 days leading up to egg laying by Algonquin females; Sechley et al. 2013).

In searching for environmental factors that might favor greater body masses in Anticosti gray jays we excluded abiotic factors such as latitude, temperature, and seasonality as the jay-inhabited land masses surrounding the Gulf of St. Lawrence where Anticosti is situated have climates similar to that of the island (Wilson 1971). We also reject the possibility that Anticosti gray jays are evolving toward an optimum body size following ecological release from absent competitors or predators (on adults). The only plausible competitor, the blue jay (*Cyanocitta cristata*), is very rare on Anticosti, but also on the Côte Nord and in the high-elevation areas of Gaspésie where we sampled mainland gray jays. As for predation on adults, the mainland's only important potential predators on adult gray jays, northern goshawks (*Accipiter gentilis*), sharp-shinned hawks (*Accipiter striatus*), and merlins (*Falco columbarius*), are all present on Anticosti (Ouellet 1969; Gauthier and Aubry 1996). The Quebec breeding bird atlas (1984–1989) suggested that the two *Accipiter* spp. may be less common than on the mainland but deemed merlins to be more abundant on Anticosti than in any other area of southern Quebec (Gauthier and Aubry 1996).

Anticosti does differ ecologically from the mainland, however, in two striking ways that may have a bearing on the greater body masses in the island population. First, concerning diet, berries are a prominent jay food on the mainland (Strickland and Ouellet 2011) but have been eradicated on wolfless Anticosti by the high density of introduced white-tailed deer (Huot 1982; Potvin et al. 2003; Côté 2005). In contrast, offal from thousands of starved or hunter-killed deer on Anticosti provides an important source of fat and protein not normally available to gray jays on the mainland where such sources are much rarer and are monopolized by predators and larger scavengers. We nevertheless question whether carcasses are so uniformly distributed in time and space that all Anticosti gray jays would have a sufficiently guaranteed access to them to account for the greater body masses that we observed, independent of territory, sex, or social status.

The second prominent Anticosti-mainland ecological difference is the island's conspicuous lack of mammalian nest predators. Historically, Anticosti had only five native nonvolant mammals (Cameron 1958) and, of these, the only possible predators on gray jay nests, black bear (*Ursus americanus*), and marten (*Martes americana*), are now extinct (Côté 2005). Moreover, no potential nest predators have been introduced and red squirrels (*Tamiasciurus hudsonicus*) have apparently never occurred there (Cameron 1958). Red squirrels are important nest predators in mainland boreal forests (Darveau et al. 1997; Boulet et al. 2000), and the importance of reducing squirrel-attracting traffic to active jay nests has been postulated as the selective force responsible for restriction of allofeeding in the gray jay to the fledgling period (Strickland and Waite 2001). It is plausible therefore that the lack of squirrels and other potential nest predators contributes to the exceptionally dense gray jay population that we and others (this study, Ouellet 1969; Gauthier and Aubry 1996) have observed on Anticosti.

Regardless of its exact cause(s), Anticosti's high gray jay density presumably engenders an unusually competitive social environment in which greater body masses may confer a significant advantage in the acquisition and defence of breeding territories (Williamson 1981; George 1987; Adler and Levins 1994). If heightened intraspecific competition accounts for the shift toward greater breeder body masses on Anticosti, one might expect even greater body masses in subordinate individuals as has been reported in several group-living northern parids, apparently as a bet-hedge against being excluded from food sources by more dominant individuals (Ekman and Hake 1990; Clark and Ekman 1995; Pravosudov et al. 1999). The greater body mass of juvenile male gray jays probably accounts for their predominance in the June intrabrood competitions that normally lead to the expulsion of all but one juvenile from the natal territory (Strickland 1991), but we have no evidence from our long-term Algonquin dataset that subordinate group members maintain greater body masses than the same-sex breeders with which they are associated (D. Strickland and D. Ryan Norris, unpubl. data). The possibility that exceptionally competitive environments may promote phenotypic elevation of body masses by subordinate individuals was nevertheless suggested on one Anticosti territory where three sibling female juveniles were retained on their natal territory for a year. This was the only case ever observed on any of our study areas where three nonbreeders remained in the family group after the normal June dispersal period (also 221 cases where one was retained and two cases where two were retained; D. Strickland and D. Ryan Norris, unpubl. data). Strikingly, the retained juveniles in this presumably exceptionally competitive situation were the heaviest females we have ever handled outside the laying period (*n *=* *279) and also remarkably close to each other in weight (range: 83.0–84.7 g). We speculate that none of the three juvenile females had a clear competitive advantage over its siblings and each may have escalated its weight in order to prevail in, or at least not lose, the (in this case stalemated) postfledging intrabrood competition that normally leads to the expulsion of subordinate juveniles from the natal territory. This example may therefore provide further support for heightened intraspecific competition being a spur to phenotypic mass increases in Anticosti gray jays.

Whatever the exact causal mechanism that has led to their greater body masses, to our knowledge, the gray jays of Anticosti Island provide the only example where, as proposed by Adler and Levins (1994), initial adherence to the island rule may be phenotypic only (and therefore not associated with genetically controlled, structurally larger body sizes). We suspect, however, that other cases may be discovered, particularly on high-latitude, recently deglaciated and colonized islands where there may have been relatively little time for genetic differentiation to occur. We encourage more genetic, morphometric, and ecological investigations of insular populations to search for further examples of early, possibly phenotypic-only adherence to the island rule.
